# Transplacental Transmission of *Leishmania infantum* as a Means for Continued Disease Incidence in North America

**DOI:** 10.1371/journal.pntd.0001019

**Published:** 2011-04-12

**Authors:** Paola Mercedes Boggiatto, Katherine Nicole Gibson-Corley, Kyle Metz, Jack Michael Gallup, Jesse Michael Hostetter, Kathleen Mullin, Christine Anne Petersen

**Affiliations:** 1 Immunobiology Program, College of Veterinary Medicine, Iowa State University, Ames, Iowa, United States of America; 2 Department of Veterinary Pathology, College of Veterinary Medicine, Iowa State University, Ames, Iowa, United States of America; 3 Laboratory Animal Resources, Iowa State University, Ames, Iowa, United States of America; 4 Department of Epidemiology, College of Public Health, University of Iowa, Iowa City, Iowa, United States of America; Institute of Tropical Medicine, Belgium

## Abstract

**Background:**

Dogs are the predominant domestic reservoir for human *L. infantum* infection. Zoonotic visceral leishmaniasis (ZVL) is an emerging problem in some U.S. dog breeds, with an annual quantitative PCR prevalence of greater than 20% within an at-risk Foxhound population. Although classically *Leishmania* is transmitted by infected sand flies and phlebotomine sand flies exist in the United States, means of ongoing *L. infantum* transmission in U.S. dogs is currently unknown. Possibilities include vertical (transplacental/transmammary) and horizontal/venereal transmission. Several reports have indicated that endemic ZVL may be transmitted vertically.

**Aims:**

Our aims for this present study were to establish whether vertical/transplacental transmission was occurring in this population of *Leishmania*-infected US dogs and determine the effect that this means of transmission has on immune recognition of *Leishmania*.

**Methodology:**

A pregnant *L. infantum*-infected dam donated to Iowa State University gave birth in-house to 12 pups. Eight pups humanely euthanized at the time of birth and four pups and the dam humanely euthanized three months post-partum were studied via *L. infantum*-kinetoplast specific quantitative PCR (kqPCR), gross and histopathological assessment and CD4+ T cell proliferation assay.

**Key Results:**

This novel report describes disseminated *L. infantum* parasites as identified by kqPCR in 8 day old pups born to a naturally-infected, seropositive U.S. dog with no travel history. This is the first report of vertical transmission of *L. infantum* in naturally-infected dogs in North America, emphasizing that this novel means of transmission could possibly sustain infection within populations.

**Major Conclusions:**

Evidence that vertical transmission of ZVL may be a driving force for ongoing disease in an otherwise non-endemic region has significant implications on current control strategies for ZVL, as at present parasite elimination efforts in endemic areas are largely focused on vector-borne transmission between canines and people. Determining frequency of vertical transmission and incorporating canine sterilization with vector control may have a more significant impact on ZVL transmission to people in endemic areas than current control efforts.

## Author Summary

Dogs are a favored feeding source for sand flies that transmit human *L. infantum* infection. Zoonotic visceral leishmaniasis (ZVL) is an emerging problem in some U.S. dog breeds, with over 20% of at-risk Foxhounds infected. Although classically *Leishmania* is transmitted by infected sand flies which exist in the United States, no role has yet been determined for vector-borne transmission. Means of ongoing *L. infantum* transmission in U.S. dogs is unknown. Possibilities include transplacental and horizontal/venereal transmission. Aims for this study were to establish whether transplacental transmission occurred in *Leishmania*-infected U.S. dogs and determine the effect of this transmission on immune recognition of *Leishmania*. This novel report describes wide-spread infection as identified by kqPCR in 8 day-old pups born to a naturally-infected, seropositive U.S. dog with no travel history. This is the first report of transplacental transmission of *L. infantum* in naturally-infected dogs in North America. Evidence that mom-to-pup transmission of ZVL may continue disease in an otherwise non-endemic region has significant implications on current control strategies for ZVL. Determining frequency of vertical transmission and incorporating canine sterilization with vector control may have a more significant impact on ZVL transmission to people in endemic areas than current control efforts.

## Introduction

Zoonotic visceral leishmaniasis (ZVL) is a vector-borne disease caused by obligate intracellular protozoan parasites of the genus *Leishmania*. In South America, dogs are the primary domestic reservoir host for ZVL [1]. Control measures for this disease are focused on vector-control and euthanasia of seropositive dogs [2], [3]. In 2000, *Leishmania infantum*, the causative agent of ZVL, was determined to be the cause of death in four Foxhounds in New York [4]. Much like human disease, signs of canine ZVL include weight loss, depression, splenomegaly, heptaomegaly, generalized lymphadenomegaly and serosanguineous nasal discharge [5]. Currently, canine ZVL in the United States is a growing problem in the Foxhound population, with an annual quantitative PCR prevalence of greater than 20% within an at-risk Foxhound population [6], [7]. Despite this and the obvious public health concerns, primary means of transmission has yet to be determined [5]. In historically-endemic regions, the sand fly is the primary vector for this disease, and although sand flies are present within the southern United States, it has not been determined if these species are competent vectors for *L. infantum* [5], [8].

There have been multiple cases of autochthonous canine ZVL in the United States. Infected dogs had not visited endemic regions nor had direct contact with other infected animals [9], [10]. Recently, two reports have demonstrated that *L. infantum* infection in endemic regions was transmitted vertically across the placenta [11] to unborn fetuses. da Silva *et. al*. described natural transplacental transmission of *Leishmania* to stillborn pups from a dog in South America [12]. Another study determined that 32% of fetuses from naturally infected dogs were PCR positive for *Leishmania* kinetoplast DNA. Although there were no gross lesions in the fetuses or placentas, low numbers of parasites were present via histology in the liver, spleen, lymph node and bone marrow [11]. In this report we describe disseminated *L. infantum* infection in multiple live day-old pups born to a naturally-infected seropositive female dog. Gross and histological findings in the dam were consistent with canine visceral leishmaniasis. While the pups were yet to acquire any gross or histologic lesions consistent with disease, *L. infantum* kinetoplast-specific qPCR analysis of multiple tissues from 10/12 pups indicated highly disseminated infection. Moreover, the dam and all pups tested had *L. infantum*-specific CD4^+^ T cell proliferative responses, suggesting an ongoing immune response specific to the parasite and not a naïve immune response. To our knowledge, this is the first determination of transplacental transmission of *Leishmania infantum* in naturally-infected dogs in North America.

## Materials and Methods

### Animals

A pregnant, seven-year old American Foxhound female was donated to Iowa State University, Department of Veterinary Pathology in March of 2009 following demonstration of seropositivity via Centers for Disease Control Indirect Immunofluorescent assay (IIF) a whole parasite-based serological method (1∶128). Three weeks after arrival, the dam gave birth to 12 pups, of which 8 were euthanized within 24 hours, and 4 were euthanized 12 weeks after birth, along with the dam. All animal use involved in this work were according to International AAALAC accreditation standards and ISU institutional IACUC approval. Animals were donated to ISU for use after signed informed consent. ISU animal facilities and programs are annually inspected and found to be above all guidelines by NIH, USDA and recently AAALAC.

### Gross and histopathology

At the time of necropsy a complete set of tissues from all animals were collected and fixed in 10% neutral buffered formalin. Tissues were routinely processed and stained with hematoxylin and eosin (H&E) for histopathologic evaluation.

### Parasites

A North American canine isolate of *Leishmania infantum*, (LIVT-2) [13], was grown to stationary phase in complete Grace's medium (Incomplete Grace's supplemented with 20% fetal bovine serum, 100 U/ml penicillin, 100 µg/ml streptomycin and 2 mM L-glutamine). Freeze-thawed whole antigen was prepared as described previously [14].

### Peripheral blood mononuclear cell (PBMC) Isolation and Carboxyfluorescein succinyl ester (CFSE) Staining

PBMC were isolated from heparinized whole blood samples using Ficoll-Histopaque 1077 (Sigma, St. Louis, MO) gradient centrifugation. Red blood cells were removed using ACK lysis buffer (0.15 M NH_4_Cl, 1.0 mM KHCO_3_, 0.1 mM Na_2_EDTA, pH 7.4). PBMC were labeled with CFSE (Molecular Probes, Eugene, OR) as described previously [15]. PBMC were washed twice in phosphate-buffered saline (PBS) and resuspended in complete medium (RPMI 1640 supplemented with 10% fetal bovine serum, 100 U/ml penicillin, 100 µg/ml streptomycin, 2 mM L-glutamine, and 25 mM HEPES buffer). PMBC were counted and adjusted to 4×10^6^/ml for further analysis.

### PBMC Proliferation Assay

CFSE-labeled PBMC (4×10^5^/well) were plated into 96-well plates and incubated with media alone, stimulated with concanavalin A (ConA) (5 µg/ml) for 4 days or with freeze-thawed, whole *L. infantum* antigen (10 µg/ml) for 7 days at 37°C with 5% CO_2_. Cells were harvested, washed in FACS buffer (0.1% albumin, 0.1% sodium azide in PBS) and labeled with PE-conjugated anti-canine CD4 antibody (Serotec, Raleigh, NC). Cells were fixed in 1% paraformaldehyde and analyzed using the FACSCanto flow cytometer (BD Pharmingen, San Diego, CA). Data was analyzed using FlowJo software (Tree Star Inc., Ashland, OR).

### Serology and real-time kqPCR

Serum samples were collected from all animals, stored at −20°C and sent to the Centers for Disease Control and Prevention for IIF testing for antibodies to *Leishmania spp.* as previously described [16]. DNA from whole blood samples collected in heparinized tubes (BD Pharmingen, San Diego, CA) was isolated using the Qiagen blood DNA isolation kit according to manufacturer's instructions. Samples of placenta, bone marrow, liver, lymph node, lung, spleen, thymus, and umbilicus were collected individually and stored at −20°C until processed for DNA extraction similar to whole blood. DNA quality and quantity was measured using a NanoDrop spectrophotometer ND1000 (Wilmington, DE). *L. infantum* kinetoplast DNA (kDNA)-specific primers and probe F 5′-CCGCCCGCCTCAAGAC, R 5′-TGCTGAATATTGGTGGTTTTGG, (Integrated DNA Technologies, Coralville, IA), Probe 5′-6FAM-AGCCGCGAGGACC-MGBNFQ (Applied Biosystems, Foster City, CA) were used. (FAM: laser-activated reporter dye; MGBNFQ: 3′-minor-groove binder non-fluorescent quencher). BLAST analysis indicated that these primers and probe were specific for *L. infantum*. DNA from *L. amazonensis* or *L. major* parasites did not amplify using this primer and probe set. Blood DNA samples were assayed via qPCR in duplicate of three dilutions (straight, 1∶10, 1∶20) using a Stratagene Mx3005P qPCR System in a 96-well format and Platinum qPCR SuperMix-UDG Master Mix (Invitrogen, Carlsbad, CA) as previously described [7]. Results were analyzed via MxPro QPCR software version 4.01 in conjunction with Microsoft Excel.

### Parasite load determination

Ten serial 1∶5 dilutions of a carefully calibrated sample containing 10^9^ whole parasites/ml were made. 50 µl of each respective parasite dilution was subsequently spiked into 150 µl of fresh canine whole blood that was collected in heparinized tubes (BD Pharmingen, San Diego, CA) as described previously [7]. Each parasite-spiked blood sample was subsequently extracted for DNA using the Qiagen blood DNA isolation kit as above. Assuming a parasite extraction efficiency of ∼90%, 6 µl (the amount used for each qPCR reaction) of each of the ten resulting full-strength, straight (0.75-strength blood) DNA extracts were calculated to contain 1,350,000, 270,000, 54,000, 10,800, 2,160, 432, 86.4, 17.28, 3.46 and 0.69 total parasites/sample, respectively, while the 1∶2.4-diluted series (to give a 1∶10 dilution in well) was calculated to contain 562,500, 112,500, 22,500, 4,500, 900, 180, 36, 7.2, 1.44 and 0.29 total parasites, respectively. Each of the 20 samples were analyzed by qPCR in triplicate using a Stratagene Mx3005P qPCR System in a 96-well format and Platinum qPCR SuperMix-UDG Master Mix (Invitrogen, Carlsbad, CA) as described previously. 1∶10-diluted DNA samples yielded the most consistent qPCR results and longest dynamic range. Each *L. infantum* parasite on average contains from 1 to 7.4 copies of the kinetoplast DNA sequence targeted by our qPCR primer and probe set [17]. The limit of detection (LOD) of this assay was determined to be ∼7.2 kinetoplast copies, or 1 parasite, per qPCR reaction. This represents an LOD range of 400–3,000 parasites per ml of canine blood, dependent on kinetoplast copy number and parasite stage. Parasite load was calculated as follows: initial copies per reaction = E_AMP_^(b – Cq)^, Copies_o_ = 2.0255^(46.764 – Cq)^, where E_AMP_ is the exponential amplification, as determined by the slope of the standard curve (E_AMP_ = 10^−1/m^), b = y intercept or limit of detection cycle threshold and Cq is the cycle of detection of a particular sample. This equation was directly applied to the Cq derived from each diagnostic/clinical dog blood sample extracted for DNA and subjected to kqPCR.

## Results

### Gross and histopathology

Twelve puppies were born to a *L. infantum*-naturally infected female Foxhound. At the time of birth the dam was both serologically (1∶128) and kqPCR positive for *L. infantum*. On histopathologic evaluation of the dam, findings in the bone marrow, liver and spleen were consistent with lymphoplasmacytic and histiocytic inflammation, likely due to disseminated visceral leishmaniasis. Rare amastigotes were noted within the spleen and liver. Of the twelve puppies, eight (1–8) were euthanized within 24 hours of birth and 4 (A–D) were euthanized 12 weeks after birth. All animals were submitted for necropsy. Gross findings indicated none of the first eight pups had any gastric or intestinal contents beyond amniotic fluid and scant meconium suggesting the pups had not yet suckled, ruling out the possibility of transmammary transmission. No signs of clinical leishmaniasis were noted in the pups.

### qPCR analysis for *L. infantum*

**Figure 1:**
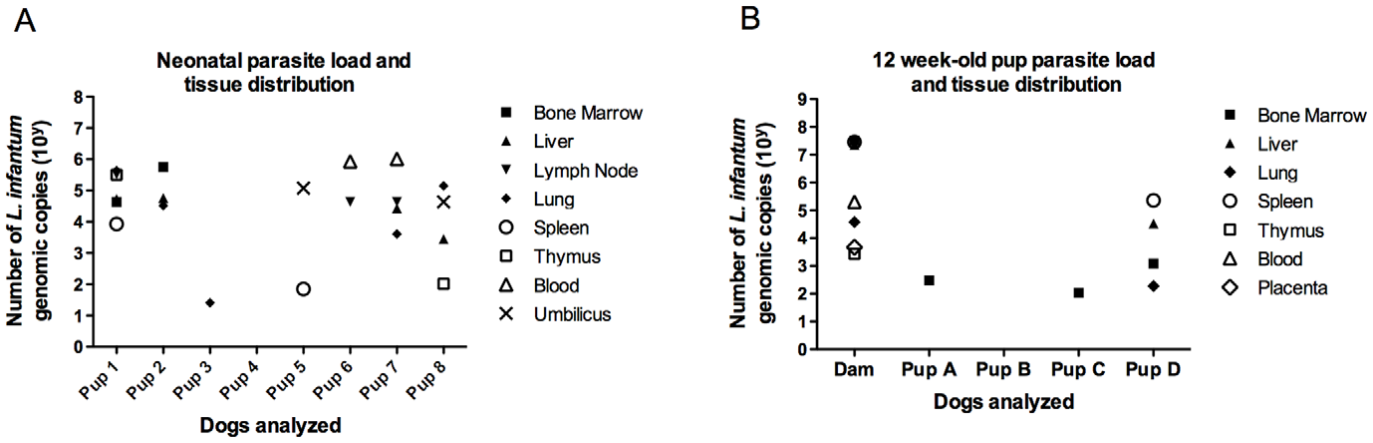
Disseminated *L. infantum* infection in pups born to an infected female. Tissue samples from target organs, as indicated, were collected at necropsy. DNA was isolated from 1 g of tissue using the QIAGEN DNA isolation kit. DNA was then analyzed via quantitative RT-PCR for the presence of *L. infantum* kinetoplast DNA. Data indicates detection of parasites in each organ and the number of *L. infantum* kinetoplast genome copies for (A) tissues from neonatal pups, 1–8, euthanized within 24 hours of birth, and (B) pups A–D euthanized at 3 months of age and the dam.

Samples from various tissues were collected during necropsy and analyzed for the presence of *L. infantum* kinetoplast DNA via qPCR. Seven of the eight puppies euthanized 24 hours after birth were positive for *L. infantum* in at least one tissue tested. Most pups, 1–2 and 5–8, showed systemic disseminated infection as the parasite was detected on multiple tissues (Figure 1A). All tissues tested from the dam were positive for *L. infantum* DNA, including the placenta, indicative of disseminated visceral infection (Figure 1B). kqPCR analysis of dogs euthanized at 12 weeks demonstrated systemic parasite dissemination only in 1 out of 4 the pups, pup D (Figure 1B). Pups A and C tested positive for *L. infantum* only in bone marrow, and pup C was not kinetoplast-qPCR positive on any of the tissues analyzed (Figure 1B). In addition, pups that tested positive for *L. infantum* via kqPCR also had a high number of parasite genomic copies (Figure 1A and B) indicative of high parasite loads in these tissues. These data suggest that not only were the puppies infected transplacentally, but also that the parasite is able to disseminate systemically *in utero* leading to high parasite loads in multiple tissues.

### *L. infantum*-specific CD4^+^ T cell response

**Figure 2:**
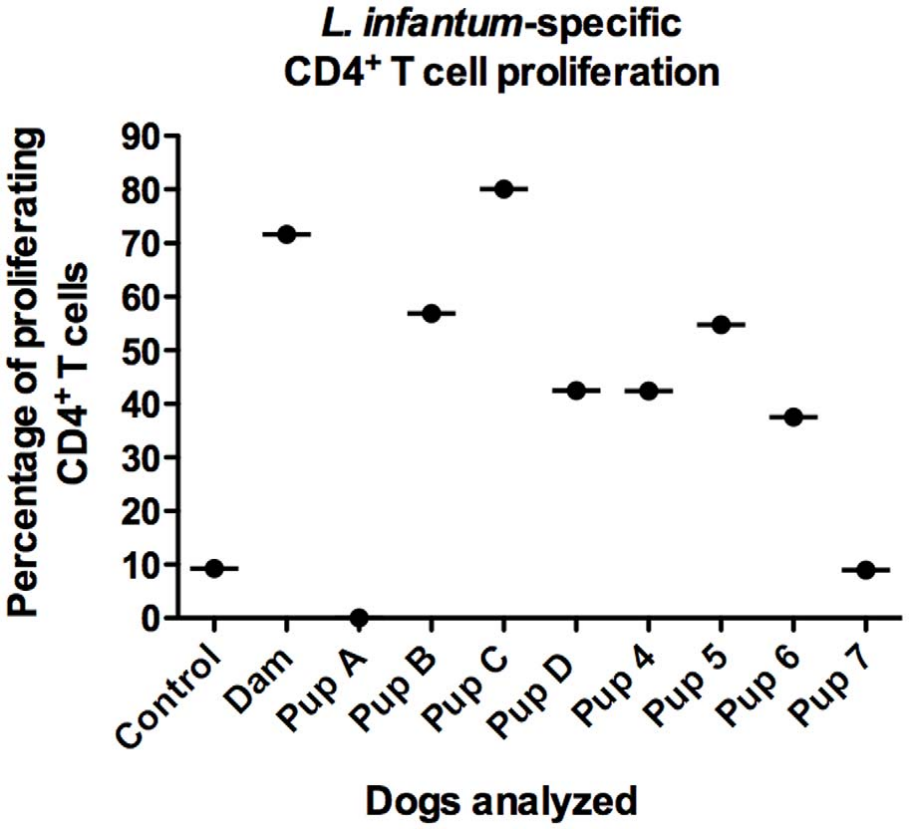
*L. infantum*-specific CD4^+^ T cell proliferative responses in vertically-infected pups. Peripheral blood mononuclear cells (PBMC) were isolated from blood samples from neonatal pups (4–7), 3-month-old pups (A–D) and the dam. PBMC were stained with CFSE and cultured in the presence of *L. infantum* freeze-thawed antigen. PMBC were harvested at day 7 and the CD4^+^ T cell proliferation response was assessed via flow cytometry. Shown are the percentages of proliferating CD4^+^ T cells in response to *L. infantum* antigen.

Whole blood samples from the dam, pups 4–7 and A–D were collected prior to euthanasia. Peripheral blood mononuclear cells (PBMC) were isolated, stained with CFSE, and stimulated with concanavalin A (ConA), *L. infantum* antigen, or were left untreated. PMBC were analyzed for CD4^+^ T cell proliferation via flow cytometry. CD4^+^ T cells from all dogs proliferated in response to stimulation with ConA, indicating that the CD4^+^ T cell compartment was not mitogenically deficient (data not shown), as previously indicated [7]. In response to *L. infantum* antigen stimulation all dogs excluding pups A and 7, had strong antigen-specific CD4^+^ T cell proliferative responses (Figure 2). These data suggest that despite likely *in utero* transmission, these pups were able to mount antigen-specific adaptive immune response at birth and were neither naïve nor immune-tolerant to *L. infantum* antigen.

## Discussion

Vertical transmission of *L. infantum* has been previously demonstrated in experimentally-infected beagles and mice, and in naturally-infected dogs in endemic areas [11], [12], [18], [19]. In the last four years *L. infantum* kqPCR incidence in kennels with clinical visceral leishmaniasis in the Midwest has remained at 12% (Petersen unpublished data) despite de-population efforts of seropositive animals. This suggests that ongoing disease transmission is occurring within the Foxhound population. Although leishmaniasis is classically described as a vector-borne disease, alternative modes of transmission including horizontal (via direct blood to blood or sexual contact) [20] and vertical (transplacental or transmammary) transmission are likely to have a role during ZVL transmission [21]. Our study demonstrates that vertical transmission of *L. infantum* occurs with a high penetrance demonstrated in one litter within the Foxhound population in the United States. If there is not a competent vector species, vertical transmission may be a primary mode of canine *L. infantum* transmission in the U.S.

We demonstrate the presence of *L. infantum* parasite kinetoplast DNA within various tissues including the placenta of an infected Foxhound dam. Given the high blood flow through the placenta during pregnancy, *L. infantum* amastigotes are likely to be carried to the placenta and on to the pups. With 10 out of 12 pups demonstrating *L. infantum* infection (83%) via kqPCR, transplacental infection is likely a primary mechanism of transmission in this population. Transvaginal infection has been suggested to play a role during vertical transmission of *L. infantum* in canines [22], however, the level of parasite dissemination (multi-organ), adaptive CD4+ T cell immune response to parasite antigen at birth and the high number of parasite copies detected via qPCR (Figure 1) at birth, suggests transplacental rather than transvaginal transmission, supporting previous findings [11], [18].

Histological examination of bone marrow, liver, lymph nodes and spleen tissue samples did not reveal the presence of *Leishmania* amastigotes in these pups. However, the sensitivity for identification of amastigotes in hematoxylin and eosin-stained sections is extremely low. Previous studies using parasitologic and histopathologic examination, as well as PCR which was less diagnostic than microscopic identification, have incorrectly declared that vertical transmission does not occur after infection with *L. infantum chagasi* [23]. We suggest that using well tested kqPCR in conjunction with stringent standard curves provides a more sensitive method of detection. Parasites were detected via kqPCR in all shown tissues (Figure 1), consistent with the pattern of *L. infantum* infection observed in adult canines.

Four pups were euthanized 12 weeks after birth and their tissues were analyzed histologically and via qPCR. Three of the four animals were positive for *L. infantum* in at least one tissue, but only one demonstrated disseminated infection, as kinetoplast DNA was detected in multiple tissues (Figure 1A). When compared to pups that were euthanized right after birth, there appears to be less disseminated infection and a lower parasite load in older pups. We postulate that this could be a result of differential parasite transmission among the pups, and/or immune-mediated control of *L. infantum* infection in the months following birth. While we cannot rule out the possibility of differences in parasite transmission, analysis of the *L. infantum*-specific CD4^+^ T cell response in infected animals indicates neonates and pups are all able to mount antigen-specific responses against the parasite (Figure 2, pups 4–7). While exposure to *L. infantum* antigen *in utero* could have led to the development of immunological tolerance [24], our data indicates these pups are responsive to *L. infantum* antigen and possibly able to clear the parasite from detection in many tissue sources. This may be supported by our findings that pups euthanized 12 weeks after birth show decreased parasite dissemination and parasite numbers within infected tissues (Figure 1B). To our knowledge this is the first description of a neonatal immune response to *L. infantum* infection.

In North America, four species genus *Lutzomyia* sand flies feed on mammals. *Lutzomyia anthorphora* and *Lu. diabolica* are found in Texas, and *Lu. cruciata* is found in Florida and Georgia [16]. *Lu. diabolica*, as isolated in Texas, has been shown to be infected with *Le. mexicana*, and is likely to transmit cutaneous leishmaniasis in this region [25]–[27]. *Lu. shannoni* has been identified in Alabama, Arkansas, Delaware, Florida, Georgia [28], Louisiana, Mississippi, North Carolina, South Carolina, New Jersey [9] and recently into the Midwest in Kentucky [29] and Ohio [30]. Experimental infection identified that *Lu. shannoni*, as found in South America, fed on clinically ill *Le infantum*-infected dogs became infected with *Le. infantum* [8]. Attempts have been made to find *Le. infantum*-infected sand flies in the environment of U.S. dog kennels without success [9], [16], [21]. In endemic areas the frequency of *Leishmania*-infected sand flies may range from only 0.2–1% however this frequency may also be as high as 5–7% [31]–[33]. Many entomologists believe that sand flies are playing a yet to be determined role in transmission of *Leishmania* in this country. Sand fly transmission of canine visceral leishmaniasis in the United States is a frightening possibility, but until parasite infection is found in domestic sand flies, and in the absence of human cases or cases in other breeds of dogs co-housed with Foxhounds, such transmission thankfully appears to be the exception and not the rule.

The data presented here poses new challenges and considerations for the control of ZVL transmission. Disease prevention methods that solely target the vector may not be sufficient to control canine disease dissemination. In support of this, studies focused on the effect of collar or topical insecticides to prevent ZVL transmission do not observe transmission reduction below 4% [34], [35] suggesting that infection may be maintained within a population through vertical transmission despite vector control methods. Altogether our data demonstrates for the first time vertical transmission of ZVL in North American dogs. Without evidence of a competent vector, we propose that vertical transmission may be a main mechanism for autochthonous *L. infantum* dissemination in the United States Foxhound population.
